# A Nurse-Led Care Delivery App and Telehealth System for Patients Requiring Wound Care: Mixed Methods Implementation and Evaluation Study

**DOI:** 10.2196/43258

**Published:** 2023-08-23

**Authors:** Cati G Brown-Johnson, Anna Sophia Lessios, Samuel Thomas, Mirini Kim, Eri Fukaya, Siqi Wu, Samantha M R Kling, Gretchen Brown, Marcy Winget

**Affiliations:** 1 Evaluation Sciences Unit, Division of Primary Care and Population Health Department of Medicine Stanford University School of Medicine Palo Alto, CA United States; 2 PocketRN Palo Alto, CA United States; 3 Division of Vascular Surgery Vascular Medicine Section Stanford University School of Medicine Stanford, CA United States; 4 Office of the Chief Nursing Informatics Officer Nursing Innovation & Informatics Stanford Medicine Stanford, CA United States

**Keywords:** nursing, telehealth, telemedicine, follow-up, wound care, capacity building, mobile phone

## Abstract

**Background:**

Innovative solutions to nursing care are needed to address nurse, health system, patient, and caregiver concerns related to nursing wellness, work flexibility and control, workforce retention and pipeline, and access to patient care. One innovative approach includes a novel health care delivery model enabling nurse-led, off-hours wound care (PocketRN) to triage emergent concerns and provide additional patient health education via telehealth.

**Objective:**

This pilot study aimed to evaluate the implementation of PocketRN from the perspective of nurses and patients.

**Methods:**

Patients and part-time or per-diem, wound care–certified and generalist nurses were recruited through the Stanford Medicine Advanced Wound Care Center in 2021 and 2022. Qualitative data included semistructured interviews with nurses and patients and clinical documentation review. Quantitative data included app use and brief end-of-interaction in-app satisfaction surveys.

**Results:**

This pilot study suggests that an app-based nursing care delivery model is acceptable, clinically appropriate, and feasible. Low technology literacy had a modest effect on initial patient adoption; this barrier was addressed with built-in outreach and by simplifying the patient experience (eg, via phone instead of video calls). This approach was acceptable for users, despite total patient enrollment and use numbers being lower than anticipated (N=49; 17/49, 35% of patients used the app at least once beyond the orientation call). We interviewed 10 patients: 7 who had used the app were satisfied with it and reported that real-time advice after hours reduced anxiety, and 3 who had not used the app after enrollment reported having other resources for health care advice and noted their perception that this tool was meant for urgent issues, which did not occur for them. Interviewed nurses (n=10) appreciated working from home, and they reported comfort with the scope of practice and added quality of care facilitated by video capabilities; there was interest in additional wound care–specific training for nonspecialized nurses. Nurses were able to provide direct patient care over the web, including the few participating nurses who were unable to perform in-person care (n=2).

**Conclusions:**

This evaluation provides insights into the integration of technology into standard health care services, such as in-clinic wound care. Using in-system nurses with access to electronic medical records and specialized knowledge facilitated app integration and continuity of care. This care delivery model satisfied nurse desires for flexible and remote work and reduced patient anxiety, potentially reducing postoperative wound care complications. Feasibility was negatively impacted by patients’ technology literacy and few language options; additional patient training, education, and language support are needed to support equitable access. Adoption was impacted by a lack of perceived need for additional care; lower-touch or higher-acuity settings with a longer wait between visits could be a better fit for this type of nurse-led care.

## Introduction

### Nursing State of the Profession: Wellness and Retention Risks

The profession of nursing has remained largely in-person and hands-on, even as COVID-19 and other 21st-century workplace stressors have pushed other professions to explore remote working. Investigations into the future of work for nursing identified nurse priorities in 2021 related to well-being, flexibility, new delivery models (eg, via digital tools), and pipeline development [1]. Each of these priorities could be addressed by adapting nursing to remote working solutions. In addition, expanding the reach of nursing services through remote work could improve access to care for patients and their informal caregivers.

Without adaptation, nursing is on the brink of a crisis driven by burnout and lack of attention to nurse well-being. In 2022, a total of 32% of the nursing workforce reported wanting to leave their current positions the next year owing to factors such as insufficient staffing, workload, and emotional toll [1,2]. In addition to a general shortage of nurses, nurses have increased responsibilities beyond providing clinical care, including patient communication and time spent charting, which can lead to burnout and adversely impact their wellness [3].

Work flexibility is a major contributor to nursing wellness and burnout prevention, which could be facilitated by allowing remote work solutions. Empowering nurses to control their work can increase job satisfaction and care quality and reduce burnout [4,5]. Remote work, such as telehealth delivered by nurses, lends itself to control and flexibility, which job seekers report valuing—specifically control over where they are working [6]. Providing remote work options could address pipeline concerns for health care systems by making individual positions more attractive or accessible to nurses who are retiring, injured, or on leave, thereby increasing the longevity of their nursing career.

### Telehealth in Specialized Care Settings

Telehealth, in the form of video visits implemented in specialized care settings, has rapidly expanded in response to the COVID-19 pandemic [7-9]. The advantages of telehealth include additional patient education, convenience, and triage [3,7,9,10]. Telehealth provides opportunities for patient coaching and education outside of clinic visits. For patients who live far from health care services, access to specialized care and triage for health care concerns can be convenient and benefit the system as well as patients [7,9]. Wound management can impose substantial costs on both patients and health systems owing to the need for frequent home health visits for dressing changes and the high expenses associated with caring for wound complications [11]. Specifically for wound care, telehealth services delivered by nurses may predict reduced acute care use [10,12-15].

Limitations of current telehealth care include the lack of tactile assessment, potential poor resolution and image quality, and additional time and effort needed to instruct patients (eg, on obtaining clinically useful images) [7,9]. Indeed, some successful telehealth interventions have not allowed nurses to work remotely or patients to receive on-demand care [12,13,15]. For example, telehealth may require an in-person nurse to be present for tactile assessment or may require patients to travel to facilities with specialized equipment for capturing wound images [12,13,15]. Nurse-led telehealth that is appropriately integrated into existing in-clinic care models could address these issues, maintaining the convenience advantage of telehealth for both patients and nurses, empowering patients with just-in-time coaching and education, and potentially triaging any wound care concerns before more serious complications occur.

### Nurse-Led Telehealth

Innovative solutions to nursing care are needed to address nurse, health system, patient, and caregiver concerns related to nursing wellness, work flexibility and control, workforce retention and pipeline, access to patient care. One such innovative approach is a nurse-led care delivery app that mobilizes generalist and specialty nurses working remotely. Applied to home-based chronic wound care, the app and integration (PocketRN) constitute a nurse-led telehealth care delivery approach that was designed to connect patients or caregivers with nurses when they needed help or advice on demand or through proactive scheduled video visits supporting care plan adherence, coaching, and education. Prior research has demonstrated positive changes in patient outcomes related to telehealth [16-19]. While we await specific patient and caregiver outcomes that are currently in process (not reported herein), we focused our attention on process implementation, that is, how this intervention was initially implemented and functioned in practice [20].

### Objective

This pilot study aimed to evaluate the adoption, acceptability, clinical appropriateness, and feasibility of app-based telehealth delivered by nurses for home wound care from the perspectives of both nurses and patients [20]. Specifically, we aimed to (1) identify and address early problems in implementation and functionality; (2) assess implementation science outcomes of feasibility, adoption, acceptability, and clinical appropriateness in the wound care context; and (3) inform scalability and the next steps in rolling out the intervention.

## Methods

### Nurse-Led, App-Based Telehealth Care Delivery Intervention

This nurse-led telehealth care delivery model was established in 2021 in Palo Alto, California, in the form of an app-based care platform. The app, PocketRN, was developed using a human-centered design by a team of physicians, nurses, engineers, and businesspeople brought together through the Stanford Biodesign program to facilitate on-demand and proactive access to nursing care. The participating nurses conducted video visits from their homes. The patients enrolled in the program were introduced to the app by a research coordinator. A few weeks into the study, the intervention was modified to have participating patients attend an orientation call where they were introduced to the platform by a support staff member and met with a nurse for their first nurse visit on the platform. When a patient had a health care question or concern, they logged onto the app to request a video visit with a nurse. Patients with a question or concern requiring specialty experience were matched by the platform to either a general float or a Wound, Ostomy, and Continence (WOCN)–certified nurse based on the clinical context of the question or concern. Upon notification, the nurse reviewed patient medical records and met with the patient using the video function within the platform, typically within a few minutes of the visit request. In addition, PocketRN staff could schedule proactive meetings with patients at times determined by clinicians to be clinically relevant (ie, 48 hours after discharge). The video function of the app allowed patients and nurses to see each other, integrate caregivers into the interaction, and show relevant visuals, such as wounds. Nurses in the study had access to patients’ electronic medical records (EMRs), which enabled them to see medical history, clinic after-visit summary, and other pertinent medical information as well as document their clinical encounters. The PocketRN staff worked with the clinical providers in the Stanford Advanced Wound Care Center (AWCC) to develop education, training, and escalation protocols for the nurses to support their remote work and top of license practice.

### Pilot Study Setting: Care at the Stanford AWCC

The AWCC provides multidisciplinary wound care to patients with wounds that do not heal or respond to standard treatment in 30 days, which includes conditions related to artery disease, diabetic foot ulcers, surgical wounds, and tissue damaged by various forms of cancer therapy [21]. Patients at the AWCC have scheduled weekly in-person visits, which include wound management with highly trained specialty clinicians and direct care (ie, wound debridement, revascularization procedures, and bandage and dressing changes). The AWCC model has proven to reduce the risk of major amputation by enabling more aggressive and effective limb salvage [21]. Similar to most specialty care clinics, when patients call the clinic outside of operational times (8 AM to 5 PM Monday to Friday), they are prompted to call back during business hours. Patients who call the hospital operator outside of operational times are routed to speak with a clinician on call for the associated specialty department. The on-call providers commonly cover calls for a large pool of patients and have numerous responsibilities that may create capacity constraints depending on the level and involvement of patient care needs. Communication with on-call providers is typically telephonic in nature, and patients needing immediate physical examination are urged to go to the emergency department. Patients may also use their MyChart application to send messages directly to their assigned provider and typically receive responses to questions within a matter of days. Patient calls that are clinical in nature made to the AWCC during hours of operation are responded to by any of 3 to 4 nurses who make time in between a busy clinic schedule to call patients back.

### Using a Nurse-Led Telehealth Care App-Based Delivery Model for Wound Care

Wound care was identified as a particularly promising clinical application to implement a nurse-led telehealth care delivery model because of the following factors: (1) frequency of wound care-related concerns in the home setting, (2) complexity of care and the level of specialty nursing experience needed, and (3) videos allowing nurses to more fully assess the wound compared with telephonic communications. Other reasons included a potential pool of qualified WOCN nurses, and a large pool of general float nurses available to participate. The app was available to patients daily from 4 PM to midnight. General float nurses who were matched to patients had the option to escalate care to a WOCN nurse on demand within the platform. If a change in care was considered owing to the video visit, this was communicated back to the patient’s clinicians at AWCC through EMR direct messaging, and if needed, their next appointment at the AWCC was rescheduled for earlier. All video visit encounters were documented in the EMR using a note template developed with the AWCC for this purpose. If any urgent or emergent health care issues requiring immediate intervention were disclosed, patients were referred to the nearest emergency department.

### Patient Recruitment and Enrollment

Patients receiving wound care at AWCC who fit the criteria of being aged 18 to 99 years, living at home, and having English proficiency or a caregiver proficient in English were recruited to participate in the study by a study coordinator from July 2021 to February 2022. The follow-up assessment of patients’ use of this platform ended on April 12, 2022. Patients who consented to participate were instructed via phone call or emailed instructions on accessing and signing into the platform.

### Ethics Approval

This pilot study was reviewed and approved by the institutional review board of Stanford University (IRB-60508).

### Informed Consent and Participation

Patient participants were consented before enrollment into the study by a research assistant, who ensured that the patients could articulate an understanding of the procedure, any follow-up, and risks and benefits associated with the study. All collected data were deidentified before analysis and stored in databases with Health Insurance Portability and Accountability Act–secured protection. The patients were provided with a US $25 Amazon gift card after completing a semistructured interview.

### Nurse Recruitment and Training

Part-time and per diem nurses who worked at AWCC or were part of the Stanford nursing general float pool were recruited to participate in the pilot study. Recruitment was performed through an opt-in invitation by nurse managers. All participating nurses received training in providing app-based care, logistics of participation in the trial, and use of the PocketRN platform. The general float pool nurses received wound care training before any interactions with patients on the platform to build capacity for the appropriate assessment of escalation. Escalation protocols were developed in collaboration with a group of nurses, advanced practice providers, and physicians with experience in performing wound care at the Stanford AWCC to ensure that patients received the appropriate level of expertise and care at the right time. For instance, calls were escalated from the general float nurses to the WOCN nurses if the patients had more complex issues. The escalation protocols also included red flag situations that triggered immediate in-person evaluation or follow-up. Nurses used loaned Stanford-encrypted laptops to conduct the visits and access patients’ medical records in a secure manner. For these telehealth visits, nurses were paid in accordance with previously agreed-upon union contract rates for a cardiac on-call nurse, which was determined to be a similar on-call service.

### Pilot Study Methods and Outcomes

**Figure 1:**
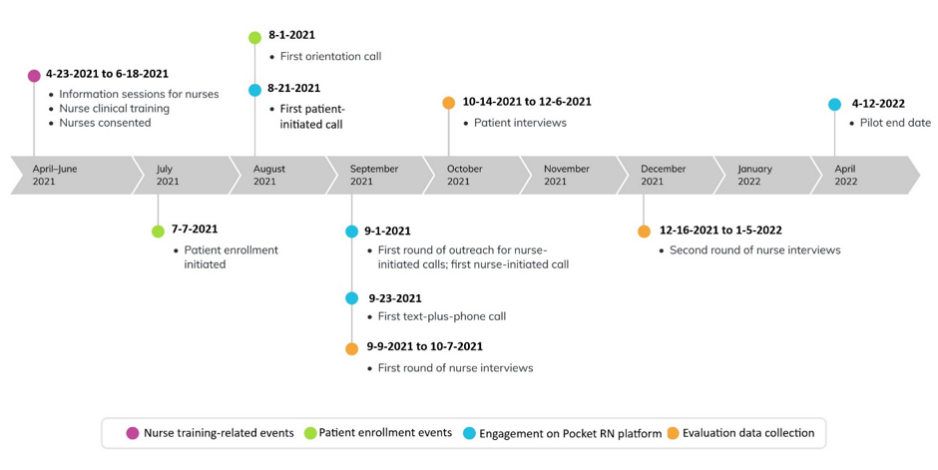
Pilot study timeline.

We conducted a prospective mixed methods evaluation that focused on implementation outcomes. Figure 1 depicts the timeline of key study events, including recruitment, data collection, and changes to the intervention made based on participant feedback and early experience. The primary outcomes, definitions, and data sources of each outcome are listed in Table 1. We assessed the adoption, acceptability, clinical appropriateness, and feasibility of the nurse-led telehealth care delivery model. Qualitative data included semistructured interviews with nurses and patients and a clinical documentation review. Quantitative data were obtained from the app and included use of the app (adoption, clinical appropriateness, and feasibility) and brief end-of-interaction satisfaction surveys collected from nurses and patients, which fell into the following outcomes: acceptability, clinical appropriateness, and feasibility.

**Table 1:** Implementation outcomes, definitions, and data sources.

Outcome	Definition	Data type and source
Adoption	Uptake of app	Quantitative: app use data (ie, from PocketRN)
Acceptability	Perception that the intervention is agreeable, palatable, and satisfactory	Qualitative: nurse interviews and patient interviews Quantitative: in-app survey
Clinical appropriateness	Perceived fit or compatibility of the intervention within this setting and for different groups of patients	Qualitative: nurse interviews and patient interviews Quantitative: in-app survey and app use data
Feasibility	Extent to which an innovation can be successfully used or carried out within a given agency or setting	Qualitative: nurse interviews and patient interviews Quantitative: in-app survey and app use data

### Patient and Nurse Interviews: Eligibility, Recruitment, and Analysis

We designed separate interview guides for patients and nurses to address the implementation outcomes of interest. All nurses who had at least 1 call on the app-based care platform were eligible and invited to participate in phone interviews, which were conducted between September 2021 and January 2022 (Figure 1).

All patients who enrolled in the study between July and December 2021 were eligible for interviews. Not all enrolled patients used PocketRN; thus, we categorized patients based on their use of the app: (1) those who had at least 1 call with nurses and (2) those who enrolled but only used the platform during an orientation call or never used it. Each group was sampled with the goal of interviewing at least 3 stakeholders (patients or caregivers) in each group. Patients were notified that they might be contacted for interviews if they consented to participate in the study. Contact information for eligible patients was shared with the interviewers. Interviewers called the patients up to 3 times to schedule and complete the interviews. Patient interviews were conducted from mid-October to mid-December 2021 by ASL and CGB-J.

Interview transcript data were analyzed using both deductive and inductive approaches. Initially, we used a rapid qualitative method, the Stanford Lightning Report approach, to analyze transcripts of interviews to identify what was working, what needed to change, and any ideas generated by participants (conducted by ASL and CGB-J) [22]. This approach has successfully been used to inform in-process implementation [23]. With input from all authors, we further organized emergent themes around the implementation outcomes of acceptability, clinical appropriateness, and feasibility [20].

### Quantitative Data and Analysis

Patient demographic and clinical characteristics were collected at enrollment by a research assistant who reviewed the patients’ EMR and entered the data into a team-designed REDCap (Research Electronic Data Capture; Vanderbilt University) database [24].

Use data were obtained from the app and included the number of interactions, type of interaction (video or phone call), person initiating the interaction (nurse or patient), reason for initiating the interaction, result of the interaction, and duration of the interaction. An in-app survey was displayed at the end of each interaction for both the nurses and patients. Each instance of the in-app survey included 3 questions that were selected from pools of 8 questions for patients and 8 questions from nurses. The questions aimed to capture the perceptions of acceptability, clinical appropriateness, and feasibility of the PocketRN visit.

Frequencies were calculated for patient demographics, app use, and in-app survey responses for all enrolled patients and also by user status (used at least once beyond the orientation call—“active user” vs used only at orientation call—“nonactive user”). Chi-square or Fischer exact tests were used, as appropriate, to calculate *P* values to assess differences between active users and nonactive users. All analyses were performed using SAS (SAS Institute).

## Results

### Overview of Nurse and Patient Participants

A total of 20 nurses were enrolled in the study: 5 were WOCN nurses and 15 were general float nurses. In total, 49 patients were enrolled in this study. We interviewed 10 (50%) of the 20 nurses and 10 (20%) of the 49 enrolled patients; 7 (70%) of the interviewed patients were active users during the study and 3 (30%) were nonactive users.

Table 2 shows the demographic and clinical characteristics of the 49 enrolled patients by user status. The majority of patients (35/49, 71%) were men, slightly more than half (26/49, 53%) were aged ≥65 years, 53% (26/49) were White, and the preferred language for most (44/49, 90%) was English. Most patients (41/49, 84%) had only 1 wound-related diagnosis. The most common diagnoses were for a type of ulcer wound, most often venous (12/49,24%), diabetic ulcer (10/49, 20%), or pressure ulcer (10/49, 20%). In total, 90% (44/49) of the patients had ≥1 comorbidities; the most common comorbidities were diabetes (15/49, 31%), peripheral vascular disease (14/49, 29%), and cancer (13/49, 27%).

**Table 2:** Demographic and clinical characteristics of the enrolled patients by user status (N=49).

Characteristics				Enrolled, n (%)		Active user (n=17), n (%)		Nonactive user (n=32), n (%)		*P* value	
**Demographic characteristics**											
	**Age (years)**										.23
		<65	23 (47)		6 (35)		17 (53)				
		≥65	26 (53)		11 (65)		15 (47)				
	**Gender**										.19^a^
		Man	35 (71)		10 (59)		25 (78)				
		Woman	14 (29)		7 (41)		7 (22)				
	**Race and ethnicity**										.08^a^
		Asian	7 (14)		1 (6)		6 (19)				
		Hispanic	5 (10)		0 (0)		5 (16)				
		White	26 (53)		13 (76)		13 (41)				
		Other or multiracial	11 (22)		3 (18)		8 (25)				
	**English language preferred**										.15^a^
		Yes	44 (90)		17 (100)		27 (84)				
		No	5 (10)		0 (0)		5 (16)				
	**Insurance**										.50^a^
		Medicare	28 (57)		9 (53)		19 (59)				
		Commercial	16 (33)		5 (29)		11 (34)				
		Medicaid	5 (10)		3 (18)		2 (6)				
**Clinical characteristics**											
	**Type of patient**										.99^a^
		Return	47 (96)		16 (94)		31 (97)				
		New	2 (4)		1 (6)		1 (3)				
	**Wound diagnosis^b^**										N/A^b,c^
		Venous ulcer	12 (24)		5 (29)		7 (22)				
		Diabetic ulcer	10 (20)		3 (18)		7 (22)				
		Pressure ulcer	10 (20)		3 (18)		7 (22)				
		Surgical wound	8 (16)		2 (12)		6 (19)				
		Other^d^	15 (31)		5 (29)		10 (31)				
	**Comorbidities^b^**										N/A^b^
		Type 1 or 2 diabetes mellitus	15 (31)		3 (18)		12 (38)				
		Peripheral vascular disease	14 (29)		4 (24)		10 (31)				
		Cancer	13 (27)		6 (35)		7 (22)				
		Congestive heart failure	9 (18)		5 (29)		4 (13)				
		Moderate to severe chronic kidney disease	9 (18)		3 (18)		6 (19)				
		Other^e^	9 (18)		3 (18)		6 (19)				

^a^*P* value based on Fisher exact test.

^b^Multiple responses possible; percentages add to more than 100%.

^c^N/A: not applicable.

^d^Other includes arterial ulcer, trauma, and “other ulcer or wound.”

^e^Other includes cerebrovascular accident or transient ischemic attacks, hemiplegia, paraplegia, quadriplegia, myocardial infarction, connective tissue disease, and chronic obstructive pulmonary disease.

### Adoption

Use of the nurse-led telehealth care delivery model by patients was slow early on; thus, modifications to the implementation process were made to help orient patients to the value of nurse-led telehealth care and ensure they knew how to use the platform (Figure 1). Specifically, 2 modifications were made early in the pilot study. The first was integrating an orientation call after study enrollment to coach patients on the way to use the application and to introduce patients to nurses working on the platform. The second was adding proactive scheduled nurse-initiated “check-in” calls. The orientation call was the first opportunity for patients to connect with a nurse on the platform and was a key element in patients’ use of the platform. The check-in calls occurred every other week, and most patients agreed to participate. In total, 70% (14/20) of the enrolled nurses participated in at least 1 nurse-initiated or patient-initiated interaction via PocketRN. Not all enrolled nurses were able to use the platform, as some nurses had a change in availability owing to personal reasons, taking leave, or being reassigned to other clinical areas. Six nurses were available for only a limited period during the study.

Of the 49 patients enrolled in the study, 26 (53%) registered with the app on their own or via an orientation call. In total, 35% (17/49) of the “active users” used the application at least once during the study period outside of the orientation call (Tables 2 and 3). The range of use of the 17 active users was 1 to 14 calls (on demand or check in). Of the 32 people who did not use the platform at least once beyond the orientation call, 9 (28%) had an orientation call and 23 (72%) did not, despite multiple attempts to schedule an orientation call. Interactions were primarily proactive nurse-initiated calls; 71 (81%) of the 88 interactions were nurse initiated, with calls placed to 15 (88%) of the 17 active users. In total, 19% (17/88) of the interactions were on-demand patient-initiated calls by 7 (41%) of the 17 active users (Table 3).

There were some differences between patients who used PocketRN and those who did not (Table 2), although none were statistically significant owing to the small sample size. Specifically, a larger proportion of active users than nonactive users were aged >65 years (11/17, 65% vs 15/32, 47%), women (7/17, 41% vs 7/32, 22%), and White (13/17, 76% vs 13/32, 41%). Importantly, 5 of the 5 (100%) patients who preferred a language other than English did not use the app. The most frequent type of wound for active users was a venous ulcer (5/17, 29%), whereas nonactive user wound types were split across venous, diabetic, and pressure ulcers (7/32, 22%). The most common comorbidities for active users were cancer (6/17, 35%) and congestive heart failure (5/17, 29%), whereas the most common comorbidity for nonactive users was diabetes (12/32, 38%).

**Table 3:** Characteristics of nurse-initiated check-in and patient-initiated app interactions.

			Total (n=88), n (%)	Nurse initiated (n=71), n (%)	Patient initiated (n=17), n (%)
**Adoption**					
	Total number of interactions		88 (100)	71 (100)	17 (100)
**Clinical appropriateness**					
	**Purpose of call**				
		Other wound^a^	71 (81)	67 (94)	4 (24)
		Pain or discomfort	4 (5)	0 (0)	4 (24)
		Wound dressing	4 (5)	0 (0)	4 (24)
		New or worsening wound	3 (3)	0 (0)	3 (18)
		Compression bandages	3 (3)	2 (3)	1 (6)
		Total contact cast	2 (2)	1 (1)	1 (6)
		Wound care devices	1 (1)	1 (1)	0 (0)
	**Result of interaction**				
		Issue resolved	78 (88)	68 (96)	10 (59)
		Patient advised to see provider	4 (5)	2 (3)	2 (12)
		Patient advised to go to emergency department	3 (3)	0 (0)	3 (18)
		Clinic to follow-up with patient	2 (2)	1 (1)	1 (6)
		Escalation to wound care nurse	1 (1)	0 (0)	1 (6)
**Feasibility**					
	**Technology used**				
		PocketRN app	79 (90)	62 (87)	17 (100)
		Audio only	9 (10)	9 (13)	0 (0)
	**Meeting duration (minutes)**				
		<5	6 (7)	6 (8)	0 (0)
		5-10	19 (22)	19 (27)	0 (0)
		10-14	21 (24)	18 (25)	3 (18)
		15-19	18 (20)	16 (23)	2 (12)
		20-24	9 (10)	7 (10)	2 (12)
		>25	15 (17)	5 (7)	10 (59)
	**Nurse type**				
		Wound, Ostomy, and Continence–certified nurse	34 (39)	24 (34)	10 (55)
		General float nurse	54 (61)	46 (66)	8 (45)

^a“^Other wound” was the default “purpose” for nurse-initiated check-ins. Other options were checked as appropriate if the patient indicated a need for help or advice on a specific wound-related issue.

### Acceptability

#### Overview

Table 4 describes the qualitative findings by implementation outcomes and their subthemes: acceptability, clinical appropriateness, and feasibility. Both interviews and in-app surveys indicated strong acceptability by patients and nurses, both of whom expressed satisfaction with the platform. The overall survey feedback was highly positive for both patient and nurse respondents, with little variation; almost all responses had the highest possible rating. The survey response rate for patients, however, was low; only 5 (29%) of 17 patients completed the surveys (13/88, 15% possible surveys). In contrast, all nurses who used the app (14/14, 100%) completed the survey after almost all interactions (82/88, 93%). In the interviews, nurses expressed positive acceptability of remote working and noted that training and experience with remote care were helpful in increasing their comfort with the care provided.

**Table 4:** Qualitative results by outcome and subtheme with exemplar quotes.

Outcomes and subthemes		Exemplar quotes
**Acceptability**		
	Patient satisfaction	“Well, I like because it’s somebody who is cared and interested about your worries and concerns about your medical condition. They’re somebody to talk to about your worries and concerns.” [Patient 5]
	Some patients already have support	“...my wife is actually my wound care nurse.” [Patient 12]
	Nurse perspectives and satisfaction*—*work flexibility	“The pros are I’m at home, I get to wear pajama bottoms, but my scrub top and at the comfort with my family. And a lot of my coworkers are moms, and I know that’s really convenient for them. It’s like a way to pick up extra hours without having to drive an hour to Palo Alto, because we’re float pool nurses.” [Nurse 10]
**Clinical appropriateness**		
	Access—patient perceptions	“Because if you do have a problem and you’re limited in your mobility particularly as I am—when I had the problem I actually I could have gone to the emergency room, but this was a lot better, and it avoided the need [to go to the emergency department].” [Patient 1]
	Quality of care	“The patients I talk to, even though we do discharge teaching, discharge summary stuff, as I mentioned, once they go home, those discharge summary papers, it goes somewhere else. And I usually ask them, ‘Do you still have that discharge summary education paper with you?’ Eight out of 10, they don’t have it with them. So that’s why...What they mean is they don’t follow the instruction. It’s difficult because that discharge summary instruction paper has everything, what they need to do. That means majority people are not compliant with it.” [Nurse 9]
**Feasibility**		
	Patient telehealth literacy	“It’s just I did everything on the list, and it wasn’t connecting. I was getting error messages.” [Patient 1]
	Nurse comfort	“I mean, every call is different. It’s just like the charting was the biggest thing for us, but they gave us clear templates on what to chart and it was very different than anything we’d ever done. So we were all just kind of, ‘Did you have a call? What did they talk about?’ Just trying to know what to expect and they did prepare us for it, but still it’s just something so different than any of us have ever done. ...So we have all the information we need. It was a little exciting...We wanted to stay in touch and see who had gotten calls already and how they went and what charting was like. Those are main things.” [Nurse 7]
	Equity	“Well, for now it best supports English-speaking patients with some technological know-how, living in an area with internet access.” [Nurse 5]

#### Patient Satisfaction

Patients reported that getting advice in real time after hours with a clinician helped manage their anxiety related to caring for their wound. They also reported the value of getting their questions addressed after hours, which ranged from nonurgent issues such as minor discomfort from wound site itching or general patient medical education questions to potentially life-threatening issues such as concerns about possible wound infections. Most patients noted the benefits of talking to nurses who seemed interested and available, without the perception that there was only a limited amount of time to discuss key priorities. Several patients also noted the advantages of being able to speak to a nurse who had access to their medical chart, so that nurses could access their patient history and clarify discharge instructions.

#### Some Patients Already Had Support

In contrast, some patients shared that they had not used the app or only used it a few times because they felt that their existing resources were sufficient to answer their questions. Examples of existing support included the ability to send questions to their physician through MyChart—a medical record application, having access to physicians on competing telehealth platforms, getting information from relatives who had medical backgrounds, home health care, and paid caregivers.

#### Nurse Perspectives Satisfied With Telehealth Care

Nurses reported that they liked the option to work from home, especially those who had a long commute time or were balancing schedules as at-home caregivers (eg, for children). They seemed to see their PocketRN shifts as a useful and convenient option for picking up hours and making extra money outside their in-person part-time nursing shifts. For a few nurses on medical leave or transitioning toward retirement, working on the platform was the most viable and sometimes the only option to continue to deliver patient care.

### Clinical Appropriateness

#### Overview

Most survey responses from nurses indicated that they were able to provide high-quality care via PocketRN and video visits to address patient concerns. Almost all patient responses indicated that their concerns were addressed and that they received the care they wanted. Consistent with this self-reporting, most concerns (78/88, 88%) were resolved during the call with a nurse on the platform, and 10 (11%) out of 88 calls were not resolved on the initial call (Table 3). Notably, 7 (70%) of the 10 calls that were not immediately resolved were patient initiated, whereas only 3 (4%) of the 71 nurse-initiated check-ins were escalated.

In total, 19% (17/88) of the interactions were patient initiated (Table 3). The most common reasons included pain or discomfort (4/17, 24%), wound dressing (4/17, 24%), and others (4/17, 24%). Three patient-initiated interactions occurred because the patients had new or worsening conditions. Of the 17 patient-initiated interactions, 10 (59%) concerns were resolved immediately. Other interaction results from patient-initiated calls included patients advised to go to the emergency department (3/17, 18%), patients advised to be seen in the clinic within the next few days (2/17, 12%), clinic notified to reach out to the patient (1/17, 6%), or interaction transferred from a general float nurse to a WOCN nurse (1/17, 6%). Of the 88 total calls, 34 (49%) were managed by WOCN nurses and 54 (61%) were managed by general float nurses. Of the 7 patient-initiated calls that were not immediately resolved and required escalation, the reasons were either new or worsening wound (n=2), pain or discomfort (n=2), other wound issue (n=2), or total contact cast (n=1).

Qualitative subthemes related to clinical appropriateness from analysis of the interviews fell under access and quality of care.

#### Most Patients Perceived Increased Access

Having the PocketRN platform available was helpful in increasing perceived access to care and provided psychological comfort, even for patients who did not use the platform. Patients expressed gratitude for having an “open line of communication” available after hours and getting answers to questions immediately, rather than having to leave messages and wait for a response. Patients further noted that speaking to a nurse reduced their anxiety about whether they should seek further care for their health problem. Some patients also noted that without this platform available to them, they would have had to go to the emergency department.

Some patients perceived a barrier in terms of appropriate access. This subgroup commented on not having any urgent issues and therefore not needing to use the app. Although urgent questions were one use of the platform, these patients were unaware that questions did not need to be urgent to call in.

#### Quality of Care

Nurses noted that the format of a videoconference allowed for higher quality of care than a phone call. Elements that contributed to the quality of care included patients being in their home settings without masks where they were comfortable, having caregivers present, and more time spent speaking to a nurse compared with in-clinic visits. The care being available after typical clinic hours gave caregivers the opportunity to be present even if their work schedule, COVID-19 restrictions, or physically distant location would otherwise not allow it. Both parties were able to see each other’s faces without masks, encouraging a connection. Having additional time (as compared with in clinic) allowed nurses to reinforce discharge instructions and provide patient education on wound care even for ancillary questions that might not come up in an appointment, such as how patients can bathe themselves with wounds in different locations.

### Feasibility

#### Overview

Nurse survey responses indicated that the technology enabled high-quality interaction and a positive care experience. The patients also reported that the technology was excellent with respect to meeting quality. Notably, 1 patient with 9 interactions was not able to use the video component of the technology, so instead used the audio-only (zero technology) aspect of the platform for all the interactions. This option allowed the patient to connect over a landline phone whereas the nurse used the existing procedure for connecting using the platform (Table 3).

The length of the interactions varied widely (Table 3). Patient-initiated calls ranged from 10 to >40 minutes in duration, with over half (10/17, 59%) >25 minutes. Nurse-initiated interactions tended to be shorter, with 34 of 71 (48%) between 10 and 20 minutes, but several interactions (5/71, 7%) were >25 minutes.

Three qualitative subthemes emerged from feasibility: patient telehealth literacy, nurse comfort, and equity.

#### Patient Telehealth Literacy

The patients noted the importance of having an initial orientation call to know how to connect. Nurses commented on how many participating patients tended to be older and less technologically literate. These users needed additional coaching to display their wounds on whatever device they were using and additional assistance with certain capabilities (ie, taking themselves off mute). Providing this type of support during the visit tended to increase the length of any given interaction but was necessary for appropriate use of the technology that provided high-value real-time information to the nurse compared with a telephonic interaction. Another suggestion from the patients was to make the initial patient survey used to indicate the reason for the call more user friendly, for instance, by having illustrations or definitions of frequently used terms.

#### Nurse Comfort

Nurses reported varying levels of comfort in providing app-based wound care depending on their level of experience in performing telehealth as well as their previous experience either providing wound care directly or as a general float nurse providing inpatient care. Nurses who had more wound care experience noted feeling confident that they had the resources they needed to answer patient questions and escalate care. They also noted that the questions and issues on the calls fit within their scope of practice. General float nurses expressed feeling more confident with wound care after receiving training but wanting to gain more experience with varied medical issues using the platform. One suggestion for sharing the knowledge gained through calls was to have a learning collaborative where nurses could connect and discuss lessons learned.

#### Equity

Another consideration mentioned by the nurses was that the platform worked for the patients with resources in terms of technology (ie, access to a smartphone, tablet, or computer), language (ie, English language capability), and connectivity (eg, internet access). Nurse suggestions to address the limited resources of some patients included access to interpreter services or allowing patients to call in without video. On the basis of this suggestion, a “zero-tech” option, in which the patient could phone in with no video from a landline, was used successfully for 1 patient. Nurses suggested that these features could make the app available to more diverse patients.

## Discussion

### Principal Findings

Integrating innovation and technology that can mitigate strain on nurses, patients, caregivers, and clinics is necessary in our increasingly stressed health care ecosystem. This evaluation suggests that using nurses in a new telehealth care delivery model, such as the one created by PocketRN—a platform that supports after-hours nurse-led integrated care via video telehealth—is acceptable, clinically appropriate, and feasible, with barriers in terms of initial uptake and technological literacy. Low technology literacy appeared to have a modest effect on patient adoption initially, but barriers were addressed over time by trial modifications that built in outreach from PocketRN staff to schedule proactive nurse visits every 2 weeks and orientation calls to address technology concerns and explain the proper use of the platform. Modifications to the technology owing to initial low patient uptake were intended to increase access and equity through the addition of the zero-technology solution and to promote understanding and health literacy through the addition of illustrated reasons for connecting with a nurse.

This nurse-led telehealth care delivery model was acceptable for users, despite the total enrollment and use numbers being lower than expected. Of the nonactive users, 23 were unresponsive to attempts to schedule an orientation meeting, which is a critical step in the implementation process. This rate (23/49, 47%) was higher than the previously published no-show rates [25]. There are 2 main reasons why the use of the technology was lower than expected, based on patient interviews. First, participants were patients of a wound care clinic that provided weekly visits; this high-touch case may have met most patients’ needs, and thus, many patients did not perceive a need for additional hours of care available through the app. Perhaps, patients’ needs would be higher in an alternative clinical setting.

Nurses appreciated working from home, and in terms of clinical appropriateness, they felt comfortable with the scope of practice. There was interest in additional wound care–specific training for nonspecialized nurses. The feasibility of the telehealth care delivery model was impacted by patients’ technology literacy and the availability of language options; additional patient training, education, and language support are needed to support equitable access for patients. Adoption was also impacted by a lack of perceived need for additional care; lower-touch or higher-acuity settings with a longer wait between visits could be a better fit for this type of nurse-led telehealth care.

Our implementation evaluation demonstrates that integrating a nurse-led telehealth care delivery model or other app-based tools with similar features into existing care can address some known gaps in specialty care in the home [26]. The attributes that made this telehealth care delivery model valuable included using nurses (1) from the target clinic and system (eg, wound care), (2) who had access to the electronic health record, and (3) who had specialized knowledge. This combination facilitated continuity of care and integration into clinic workflows and was seen as highly beneficial by both patients and nurses. Having a nursing telehealth option also succeeded in satisfying nursing desires for flexibility in terms of where nurses were working and allowed a few nurses who were unable to perform in-person care to provide direct patient care over the web [1]. Capitalizing on trends toward remote work, nurse-led telehealth care delivery models meet nurses where they often desire to be: at home. Finally, patients who engaged with the app reported that simply having access to nursing on demand reduced anxiety, which according to the literature could reduce major risks of postoperative wound care, such as increased pain and infection risk [27,28]. These findings suggest the benefits of intentionally integrating telehealth into clinical health services for future success.

### Limitations

This study is limited in terms of its short timeline, small number of participants, and limited generalization. As a small implementation study, these limitations can be expected (short time, few participants, and 1 organization or clinic). It is important to note that the clinical setting of the pilot study did affect both the low enrollment numbers and high no-show rate. The AWCC is a high-touch specialty wound clinic with weekly in-person appointments for all patients. Many patients did not enroll or did not attend an orientation call given the amount of time that was already being used to maintain enrollment in the AWCC. Given the diversity and complexity of the wounds and the variety of patients’ clinical and socioeconomic demographics, we aimed to study the clinical embeddedness and fit of this intervention to this diverse patient population. Therefore, patients who did not attend an orientation were not unenrolled but rather placed in a lost-to-follow-up category so that we could use any insights about that group to help specify inclusion criteria for a future study where more patients can be enrolled. Despite these typical pilot limitations, our insights and potential future applications in the growing field are valuable.

### Comparison With Prior Work

Although engaging patients within an already high-touch clinical setting was a challenge, adapting protocols early to overcome issues related to technological literacy and expectation management enabled more consistent engagement. Technology as a barrier is a known issue in terms of patient integration with health care applications. Home health technology can be challenging even for highly technology-literate individuals, as we have seen in attempts to integrate precision health into primary care [29]. Telemedicine unreadiness has been shown to be more prevalent in older individuals, with 1 study estimating unreadiness in a quarter to three-quarters of individuals aged >65 years [30]. During this study, a larger proportion of app users compared with nonusers were aged ≥65 years, and only 1 of the users required a lower-technology solution (zero technology, ie, phone). Allocating resources to walk patients through the use of the app-based care technology was important. We suggest proactive technological assistance as a must-have for any future health application. Providing an orientation to the telehealth care model was equally important in terms of the management of patient expectations, explaining when and how telehealth care models such as PocketRN can supplement in-person care. To address challenges with technology and patient activation, telehealth care models such as this will likely require up-front human resources to identify and train patients and caregivers who might need additional assistance and provide them with the technology support and context around the value of integrating app-based care into their patient journey.

Nurse-led telehealth care delivery models such as PocketRN have growth opportunities for patients who do not or are not able to engage with the platform. Patients who did not use the app platform noted existing support for their health care needs, often in the form of knowledgeable caregivers or home health. Opportunities exist for nurse-led telehealth care delivery models to interface directly with caregivers and explore partnerships with in-home care organizations. Another major gap, inequity in access owing to subgroup technology and language barriers [26], deserves further attention. In this study, some technology equity issues were provisionally addressed by replacing the video visit on the app with the audio-only zero-technology phone call solution. Of the 17 people, 1 who used PocketRN used this solution multiple times over the course of the pilot study, suggesting a high need for wound care support that could be addressed with lower-technology options. A review of low-technology, high-value care suggests designing low technology options with the following considerations: (1) emphasizing nonjudgmental patient choice for higher- or lower-technology options, (2) cataloging barriers and prepping for future higher-technology options, and (3) building out and supporting low technology (eg, audio only and even home visits as needed) [31].

### Conclusions

Nurse-led telehealth care delivery models can provide patients and caregivers after-hour access to the care they need in their home, facilitating healing with better continuity of care and reduced anxiety regarding immediate next steps for care. These models of care also provide a valuable opportunity for clinics and their nurses to reach out to patients and caregivers to revisit discharge instructions and receive coaching to be proactive about their health. This proposed system of care also addresses nurses’ needs with a novel economic opportunity to work from home. In addition to addressing general float nurses’ desires for remote work, digitally distributed care models also hold promise as an opportunity to mobilize nursing capacity that might otherwise be inaccessible, for example, nurses with injuries or unable to do in-person care (eg, owing to temporary weight-lifting restrictions). Opportunities for further development of nurse-led telehealth care delivery models such as PocketRN include expanding depth and breadth of reach, specifically by offering more low-technology solutions to increase equity of access for patients and extending app interfaces to other stakeholders beyond those with English proficiency.

## Abbreviations

AWCC: Advanced Wound Care Center
EMR: electronic medical record
REDCap: Research Electronic Data Capture
WOCN: Wound, Ostomy, and Continence

## Data Availability

The data that support these findings are available from the corresponding author upon reasonable request.

## References

[1] Berlin G, Lapointe M, Murphy M, Viscardi M (2021). Nursing in 2021: retaining the healthcare workforce when we need it most. McKinsey & Company.

[2] Berlin G, Lapointe M, Murphy M (2022). Surveyed nurses consider leaving direct patient care at elevated rates. McKinsey & Company.

[3] Shah MK, Gandrakota N, Cimiotti JP, Ghose N, Moore M, Ali MK (2021). Prevalence of and factors associated with nurse burnout in the US. JAMA Netw Open.

[4] Boamah SA, Read EA, Spence Laschinger HK (2017). Factors influencing new graduate nurse burnout development, job satisfaction and patient care quality: a time-lagged study. J Adv Nurs.

[5] Brady Germain P, Cummings GG (2010). The influence of nursing leadership on nurse performance: a systematic literature review. J Nurs Manag.

[6] Dua A, Ellingrud K, Kirschner P, Kwok A, Luby R, Palter R, Pemberton S (2022). Americans are embracing flexible work—and they want more of it. McKinsey & Company.

[7] Saliba-Gustafsson EA, Miller-Kuhlmann R, Kling SM, Garvert DW, Brown-Johnson CG, Lestoquoy AS, Verano MR, Yang L, Falco-Walter J, Shaw JG, Asch SM, Gold CA, Winget M (2020). Rapid implementation of video visits in neurology during COVID-19: mixed methods evaluation. J Med Internet Res.

[8] Vilendrer S, Patel B, Chadwick W, Hwa M, Asch S, Pageler N, Ramdeo R, Saliba-Gustafsson EA, Strong P, Sharp C (2020). Rapid deployment of inpatient telemedicine in response to COVID-19 across three health systems. J Am Med Inform Assoc.

[9] Brown-Johnson CG, Spargo T, Kling SM, Saliba-Gustafsson EA, Lestoquoy AS, Garvert DW, Vilendrer S, Winget M, Asch SM, Maggio P, Nazerali RS (2021). Patient and surgeon experiences with video visits in plastic surgery-toward a data-informed scheduling triage tool. Surgery.

[10] Grabowski DC, O'Malley AJ (2014). Use of telemedicine can reduce hospitalizations of nursing home residents and generate savings for medicare. Health Aff (Millwood).

[11] Lindholm C, Searle R (2016). Wound management for the 21st century: combining effectiveness and efficiency. Int Wound J.

[12] Bangs I, Clarke M, Hands L, Jones R, Knott M, Mahaffey W (2002). An integrated nursing and telemedicine approach to vascular care. J Telemed Telecare.

[13] Sood A, Granick MS, Trial C, Lano J, Palmier S, Ribal E, Téot L (2016). The role of telemedicine in wound care: a review and analysis of a database of 5,795 patients from a mobile wound-healing center in Languedoc-Roussillon, France. Plast Reconstr Surg.

[14] Chanussot-Deprez C, Contreras-Ruiz J (2013). Telemedicine in wound care: a review. Adv Skin Wound Care.

[15] Gagnon MP, Breton E, Courcy F, Quirion S, Côté J, Paré G (2014). The influence of a wound care teleassistance service on nursing practice: a case study in Quebec. Telemed J E Health.

[16] Patel MI, Sundaram V, Desai M, Periyakoil VS, Kahn JS, Bhattacharya J, Asch SM, Milstein A, Bundorf MK (2018). Effect of a lay health worker intervention on goals-of-care documentation and on health care use, costs, and satisfaction among patients with cancer: a randomized clinical trial. JAMA Oncol.

[17] Patel MI, Kapphahn K, Dewland M, Aguilar V, Sanchez B, Sisay E, Murillo A, Smith K, Park DJ (2022). Effect of a community health worker intervention on acute care use, advance care planning, and patient-reported outcomes among adults with advanced stages of cancer: a randomized clinical trial. JAMA Oncol.

[18] Bernacki R, Hutchings M, Vick J, Smith G, Paladino J, Lipsitz S, Gawande AA, Block SD (2015). Development of the serious illness care program: a randomised controlled trial of a palliative care communication intervention. BMJ Open.

[19] Paladino J, Bernacki R, Neville BA, Kavanagh J, Miranda SP, Palmor M, Lakin J, Desai M, Lamas D, Sanders JJ, Gass J, Henrich N, Lipsitz S, Fromme E, Gawande AA, Block SD (2019). Evaluating an intervention to improve communication between oncology clinicians and patients with life-limiting cancer: a cluster randomized clinical trial of the serious illness care program. JAMA Oncol.

[20] Proctor E, Silmere H, Raghavan R, Hovmand P, Aarons G, Bunger A, Griffey R, Hensley M (2011). Outcomes for implementation research: conceptual distinctions, measurement challenges, and research agenda. Adm Policy Ment Health.

[21] Flores AM, Mell MW, Dalman RL, Chandra V (2019). Benefit of multidisciplinary wound care center on the volume and outcomes of a vascular surgery practice. J Vasc Surg.

[22] Brown-Johnson C, Safaeinili N, Zionts D, Holdsworth LM, Shaw JG, Asch SM, Mahoney M, Winget M (2019). The Stanford Lightning Report Method: a comparison of rapid qualitative synthesis results across four implementation evaluations. Learn Health Syst.

[23] Brown-Johnson CG, Chan GK, Winget M, Shaw JG, Patton K, Hussain R, Olayiwola JN, Chang SI, Mahoney M (2019). Primary Care 2.0: design of a transformational team-based practice model to meet the quadruple aim. Am J Med Qual.

[24] REDCap. Stanford University.

[25] Kheirkhah P, Feng Q, Travis LM, Tavakoli-Tabasi S, Sharafkhaneh A (2016). Prevalence, predictors and economic consequences of no-shows. BMC Health Serv Res.

[26] Omboni S, Padwal RS, Alessa T, Benczúr B, Green BB, Hubbard I, Kario K, Khan NA, Konradi A, Logan AG, Lu Y, Mars M, McManus RJ, Melville S, Neumann CL, Parati G, Renna NF, Ryvlin P, Saner H, Schutte AE, Wang J (2022). The worldwide impact of telemedicine during COVID-19: current evidence and recommendations for the future. Connect Health.

[27] Starkweather AR, Witek-Janusek L, Nockels RP, Peterson J, Mathews HL (2006). Immune function, pain, and psychological stress in patients undergoing spinal surgery. Spine (Phila Pa 1976).

[28] Kagan I, Bar-Tal Y (2008). The effect of preoperative uncertainty and anxiety on short-term recovery after elective arthroplasty. J Clin Nurs.

[29] Brown-Johnson CG, Safaeinili N, Baratta J, Palaniappan L, Mahoney M, Rosas LG, Winget M (2021). Implementation outcomes of Humanwide: integrated precision health in team-based family practice primary care. BMC Fam Pract.

[30] Ikram U, Gallani S, Figueroa JF, Feeley TW (2020). 4 strategies to make telehealth work for elderly patients. Harvard Business Review.

[31] Alkureishi MA, Lee WW, Lenti G, Choo ZY, Benning-Shorb J, Grob R, Gaines ME, Frankel R (2021). Low-tech high-value(s) care: no patient left behind. Perm J.

